# The yeast GRASP Grh1 displays a high polypeptide backbone mobility along with an amyloidogenic behavior

**DOI:** 10.1038/s41598-018-33955-1

**Published:** 2018-10-24

**Authors:** N. A. Fontana, R. Fonseca-Maldonado, L. F. S Mendes, L. P. Meleiro, A. J. Costa-Filho

**Affiliations:** 1 Departamento de Física, Faculdade de Filosofia, Ciências e Letras de Ribeirão Preto, Universidade de São Paulo, Ribeirão Preto, SP Brazil; 2 Departamento de Química, Faculdade de Filosofia, Ciências e Letras de Ribeirão Preto, Universidade de São Paulo, Ribeirão Preto, SP Brazil; 3 Instituto Federal de Ciência e Tecnologia de São Paulo, Jacareí, SP Brazil

## Abstract

GRASPs are proteins involved in cell processes that seem paradoxical: responsible for shaping the Golgi cisternae and involved in unconventional secretion mechanisms that bypass the Golgi. Despite its physiological relevance, there is still a considerable lack of studies on full-length GRASPs. Our group has previously reported an unexpected behavior of the full-length GRASP from the fungus *C. neoformans*: its intrinsically-disordered characteristic. Here, we generalize this finding by showing that it is also observed in the GRASP from *S. cerevisae* (Grh1), which strongly suggests it might be a general property within the GRASP family. Furthermore, Grh1 is also able to form amyloid-like fibrils either upon heating or when submitted to changes in the dielectric constant of its surroundings, a condition that is experienced by the protein when in close contact with membranes of cell compartments, such as the Golgi apparatus. Intrinsic disorder and fibril formation can thus be two structural properties exploited by GRASP during its functional cycle.

## Introduction

The Golgi complex is composed of a series of cisternal membranes opposed to one another to form stacks^1^. In mammalian cells, the stacks are linked at their edges by tubules to form a ribbon-like structure^2,3^. An assay that blocks cisternal stacking in postmitotic events was the basis for the discovery of the two proteins known as Golgi Reassembly and Stacking Proteins (GRASP65 and GRASP55)^3,4^. Furthermore, other functions of GRASPs have already been reported, such as chaperoning and transport of some proteins, participation in cell apoptosis, Golgi reorientation during cell migration, unconventional protein secretion, and, during mitosis, as a possible G2/M checkpoint^5^.

GRASP structure is divided in two regions: an N-terminal half, called GRASP domain, which contains two PDZ domains^6^ and the second half (the C-terminal region), rich in proline, serine, glutamine and asparagine residues, also known as SPR domain^7–9^. The formation of the Golgi ribbon-like structure requires membrane bridging by the dimeric state of the GRASP domain^7,8^. In mammalian and Drosophila, GRASPs are tightly associated with the Golgi membranes via an N-myristoylation of the residue Gly_2_^2,10^ and, in yeasts, via an acetylated amphipathic helix^11^. The association of GRASP65 also depends on a Golgi receptor, identified as the coiled-coil protein called GM130^2^. The dual membrane association is important for the correct *trans* dimerization, a necessary step in the stack formation^12,13^.

Details of the involvement of GRASPs in membrane trafficking and other functions in mammalian cells have been reported by researchers using model organisms, such as the yeast *Saccharomyces cerevisiae*. Although *Saccharomyces cerevisiae* has the basic organization of its Golgi cisternae, only 40% of the cisternae are in stacks and the stacks are never found linked to each other^5^. This budding yeast contains a single GRASP65 homolog, known as Grh1, which localizes in compartments of the early secretory pathway^14^. Grh1 is analog to GRASP65 and forms a complex with a coiled-coil protein, Bug-1, that shares structural features with GM130. The Grh1-Bug1 complex is involved in membrane trafficking, contributes to the formation of the cis-Golgi^11^ and, although dispensable for conventional secretion, is essential for the unconventional secretion of ACBP1^15^. Furthermore, Grh1 interacts with the dimer formed by Sec. 23 and Sec. 24, protein components of the COPII coat, an event necessary for the fusion of vesicles derived from ER with Golgi membranes^11^.

Here, we present the first structural characterization of the yeast GRASP Grh1. We investigated the biophysical and biochemical features of Grh1 and the isolated GRASP domain (called here DGRASP) by circular dichroism (CD), fluorescence and optical spectroscopies, differential scanning calorimetry (DSC), computational predictions and established that Grh1 is a molten globule-like protein, making it a member of the collapsed intrinsically disordered protein (IDP) family. IDPs are proteins involved in a large set of functions and characterized by regions of high polypeptide mobility, and without a well-defined 3D structure^16,17^. These proteins have been grouped into two broad structural classes: (1) collapsed (molten globule-like) and (2) extended (coil-like and pre-molten globule-like)^18^. The structural flexibility of IDPs allows a broad functional repertoire and a number of interaction partners^19^ to act and to influence protein function in different processes, such as transcriptional regulation, translation, cellular signal transduction, and storage of small molecules^20^.

Alongside with its disorder, Grh1 also shows an unexpected feature. We report here our findings on the amyloidogenic behavior of this GRASP. They are derived from CD, fluorescence using a specific dye, and Congo Red absorbance experiments. The results obtained from this wide range of techniques led us to the conclusion that Grh1 can form amyloid-like structures in conditions that could be reasonably found in the cell. Moreover, we showed that the DGRASP, which is the most conserved region along GRASP family, is sufficient for the fiber formation. Our results suggest that this could be a general feature of GRASPs.

## Materials and Methods

### Bioinformatics Tools

The aggregation prediction was done in the AGGRESCAN server^21^, using a 5-residue window. The disorder prediction was done using the DisEMBL^22^ and the PONDR-FIT^23^ servers.

### Protein expression and purification

Genomic DNA of a strain of *Saccharomyces cerevisiae* was used as the template for PCR amplification of the gene encoding Grh1 (Gene ID: 852129) using primers Grh1F (5′-CCCGGATCCTTTAGAATAGCTAAAAACCTCGTACGG-3′) and Grh1R (5′-GGGTTCGAATTAATCAGAGGATGACTGTTTTTGTGGT-3′). The PCR reaction was carried at 94 °C for 3 min, followed by 30 cycles of 94 °C for 1 min, 50 °C for 1 min, 72 °C for 1 min and final incubation at 72 °C for 10 min. The PCR product was digested with BamHI and HindII (recognition sites underlined in the oligonucleotide sequences) and cloned into the plasmid pETSUMO. The resulting construct (pETSUMO-Grh1) was transformed into DH5α *Escherichia coli*, and the plasmid DNA was purified and sequenced. *E. coli* Rosetta (DE3) cells (Novagen, Darmstadt, Germany) transformed with pETSUMO-Grh1 were grown at 37 °C and 200 rpm agitation until reaching an OD 600 nm of 0.6 in 2 L shake flasks containing 1 L LB medium supplemented with 40 µg.mL^−1^ kanamycin and 34 µg.mL^−1^ chloramphenicol. The expression was carried out for 21 h and induced with 0.5 mM IPTG at 18 °C and 200 rpm agitation. The cells were harvested and transferred to 20 mL of lysis buffer (40 mmol.L^−1^ HEPES pH 8.0, 300 mmol.L^−1^ NaCl, and 10% Glycerol). After disruption by sonication, cell debris were removed by centrifugation, and the supernatant was applied to a nickel affinity column (Promega – Madison, USA). The column was washed with buffer containing 40 mmol.L^−1^ HEPES pH 8.0, 300 mmol.L^−1^ NaCl, 10% Glycerol supplemented with 25 mmol.L^−1^ imidazole and was eluted in the same buffer with 300 mmol.L^−1^ imidazole. The imidazole was removed by extensive washing using centrifugation in a Vivaspin column (GE Healthcare, Buckinghamshire, United Kingdom) and the sample was incubated for 3 h with recombinant ULP-1 protease followed by incubation in a nickel affinity chromatographic column to remove the SUMO protein and ULP-1 protease. The remaining contaminants were removed by size exclusion chromatography onto a Superdex 200 10/300 GL gel filtration column (GE Healthcare, Buckinghamshire, United Kingdom) in 40 mmol.L^−1^ HEPES pH 8.0, 300 mmol.L^−1^ NaCl, 10% Glycerol buffer. The purification of the GRASP domain (DGRASP) followed the same protocol, using a different reverse primer, with a stop codon at the end of the GRASP domain to exclude the SPR domain.

### Circular Dichroism (CD)

Far-UV (190–260 nm) CD experiments were carried out in a Jasco J-815 CD Spectrometer (JASCO Corporation, Japan) equipped with a Peltier temperature control and using a quartz cell with a path length of 1 mm. Grh1 was in 10 mM sodium phosphate buffer, pH 8.0 and at final concentration of 5 µM. All far-UV CD spectra were recorded with a scan speed of 50 nm/min and at time response of 1 s. Chemical stability experiments were performed in the same buffer and increasing urea concentration (0–8.0 M). To investigate the effects of solvents Grh1 was incubated in aqueous methanol (MeOH) and acetonitrile (ACN) over a range of 0–50% solvent. The spectra were averaged, baseline-corrected and smoothed with a Savitsky-Golay filter using CDTools software^24^. The processed spectra were deconvoluted by using the software Continll^25^ with database 7^26^ available in the DichroWeb analysis server^27^. The normalized root-mean-square deviation (NRMSD) goodness-of-fit parameter was always less than 0.15, suggesting that the calculated spectra are in agreement with the experimental data^28^.

### Steady-State Fluorescence Spectroscopy

Intrinsic and extrinsic fluorescence were monitored using a Hitachi F-7000 fluorimeter equipped with a 150 W xenon arc lamp. The excitation and emission monochromators were set at 2.5 nm slit width in all experiments. The protein concentration was 5 µM for Grh1 and 7 µM for DGRASP in 40 mM Hepes, 150 mM NaCl, 10% glycerol. For tryptophan fluorescence experiments, the selective tryptophan excitation wavelength was set at 295 nm and the emission spectrum was monitored from 300 up to 400 nm. The fluorescence of tryptophan across chemical denaturation was measured in increasing concentrations of urea (0–7.5 M). For the ThT experiments, 15 µM of the dye solution was used along with the protein, excited at 440 nm. For the ANS experiments, a 250 µM solution was used, with excitation at 355 nm. For the intrinsic fluorescence experiment the samples were excited at 357 nm, both at room temperature and 50 °C. The emission was monitored at 470 nm, with a 3 minutes interval between measurements.

### Congo Red Assay

The absorbance spectrum of Congo Red (CR) was monitored in the presence and in the absence of the protein, between 400 and 700 nm, with a Beckman DU 640 Uv-Vis Spectrometer. The CR was in a buffer solution as reported elsewhere^29^.

### Fluorescence Lifetime Imaging Microscopy (FLIM)

The FLIM experiments were performed in a PicoQuant MT 200 microscope. We set three different conditions: control, heating for 30 and for 90 minutes. 15 μM samples in each condition were analyzed with an excitation in 378 nm and the fluorescence lifetimes were obtained at 440 nm.

## Results and Discussions

### Sequence and structure prediction

**Figure 1:**
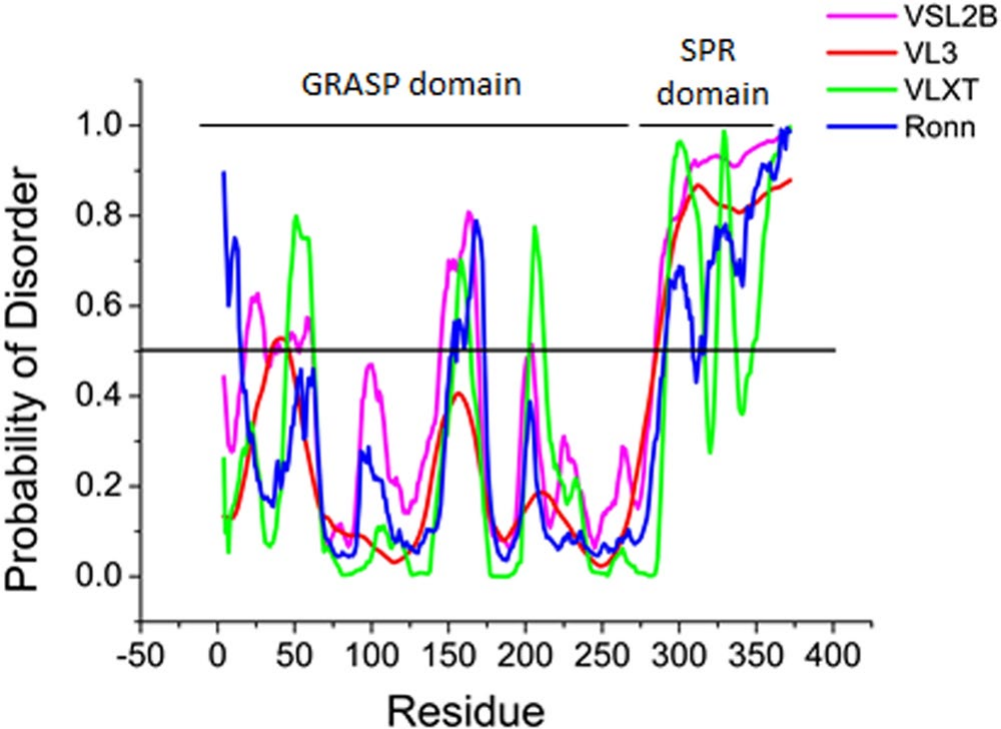
Predictions of intrinsically disordered regions in the Grh1 sequence using VSL2B (Magenta), VL3 (Red) VLXT, (Green) and Ronn (Blue). The black line indicates the threshold to be considered as a disordered region.

Grh1 is composed of 372 amino acids and the analysis of the protein family database using the Pfam program^30^ predicted that the GRASP domain, including the two PDZ subdomains, comprises residues 1 to 280, and the SPR domain extends from residue 281 to 372. In addition, the sequence-based prediction of disordered regions (Fig. 1) showed that the C-terminal domain and the central region of the PDZ subdomains (spanning 55% of the protein sequence) have high probability of being intrinsically disordered, a tendency already observed for the SPR and the PDZ subdomains within the GRASP family^31^.

### Structural Behavior in Solution

**Figure 2:**
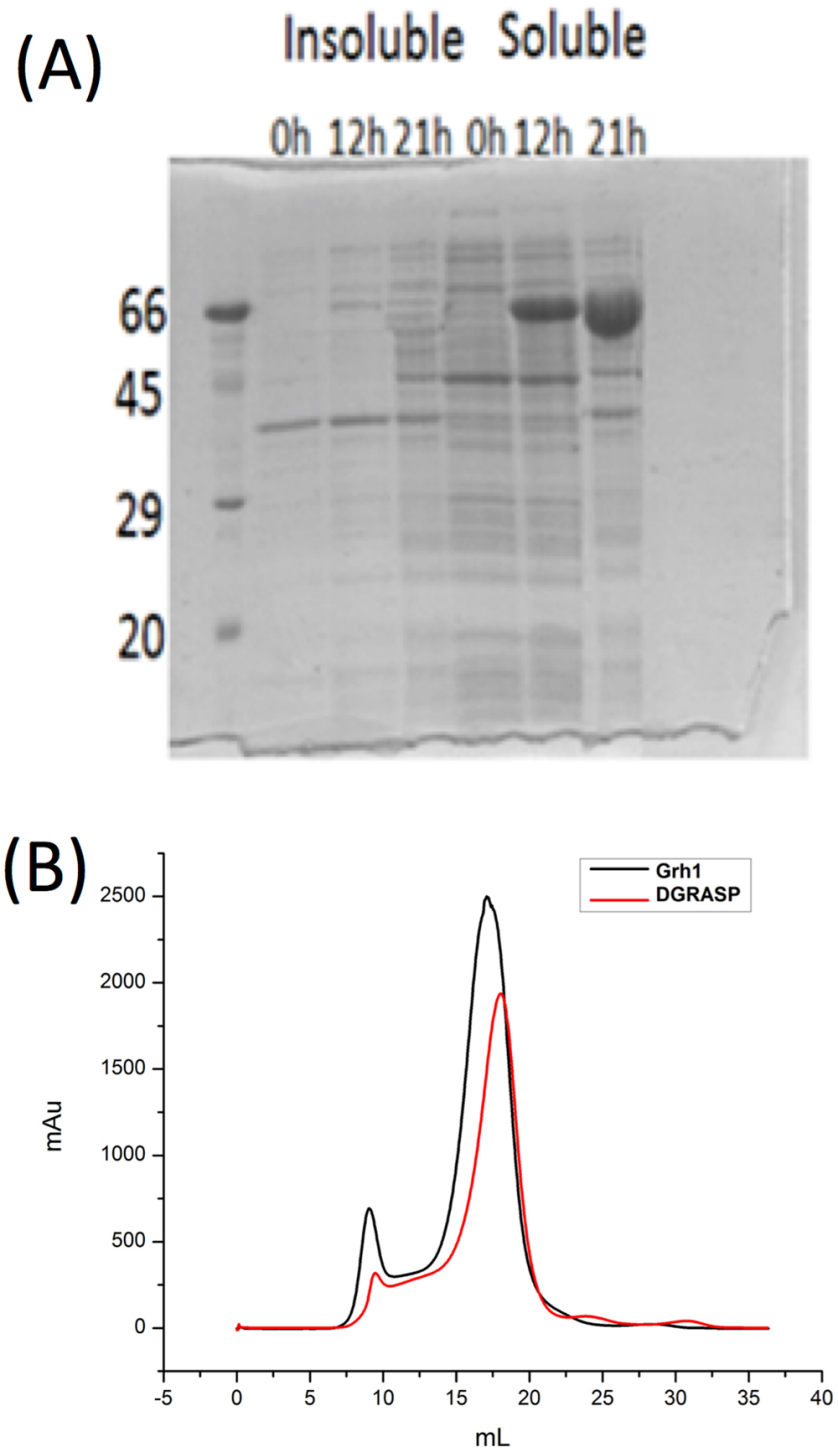
(**A**) SDS-PAGE monitoring the time course of Grh1 recombinant expression. Insoluble and soluble samples at specific times (0 h, 12 h 21 h) after IPTG induction. (**B**) Size exclusion chromatography of Grh1 and DGRASP. The first peak represents aggregates of at least 45 molecules of Grh1 and the second peak, the elution of the monomeric Grh1 and DGRASP. The pattern for the GRASP domain construct is the same observed for Grh1 (data not shown here).

Unlike GRASP55 and GRASP65^7,8^, full-length Grh1 was successfully expressed as a soluble, monodisperse protein in *E. coli* (Fig. 2A). The theoretical molecular mass of the recombinant Grh1 is 41,119 Da, but SDS-PAGE analysis (Fig. 2B) resulted in an apparent molecular mass of ca. 45,000 Da. This suggests that the amount of hydrophobic aminoacids that compose Grh1 is smaller than expected for well-structured proteins, a phenomenon similar to what was previously observed for other IDPs^32^. Size exclusion chromatography of the soluble protein on Superdex-200 column, whose result is shown in Fig. 2B, indicates an apparent molecular mass of 45,200 Da. The differences between the expected molecular mass of Grh1 and the values determined from hydrodynamic methods is likely a consequence of the not-fully globular conformation of Grh1 in solution, which has been observed for other proteins rich in disordered regions^33^, including the GRASP homologue in *C. neoformans*^31^. The chromatogram for the GRASP domain (DGRASP) is also presented in Fig. 2B. We can see that it is eluted slightly after the full-length Grh1, which is expected since DGRASP lacks the SPR domain. Based on an elution curve calibrated with molecular mass standards (Suppl. Material, Fig. S1), we conclude that Grh1 and its GRASP domain behave predominantly as a monomer in solution. This is different from the observed dimers in mammalian and rat GRASPs, which may be due to the lack, in the Grh1 primary sequence, of the residues involved in dimerization^7^ and trans-oligomerization^5^ of GRASPs in mammalian and rat.

**Figure 3:**
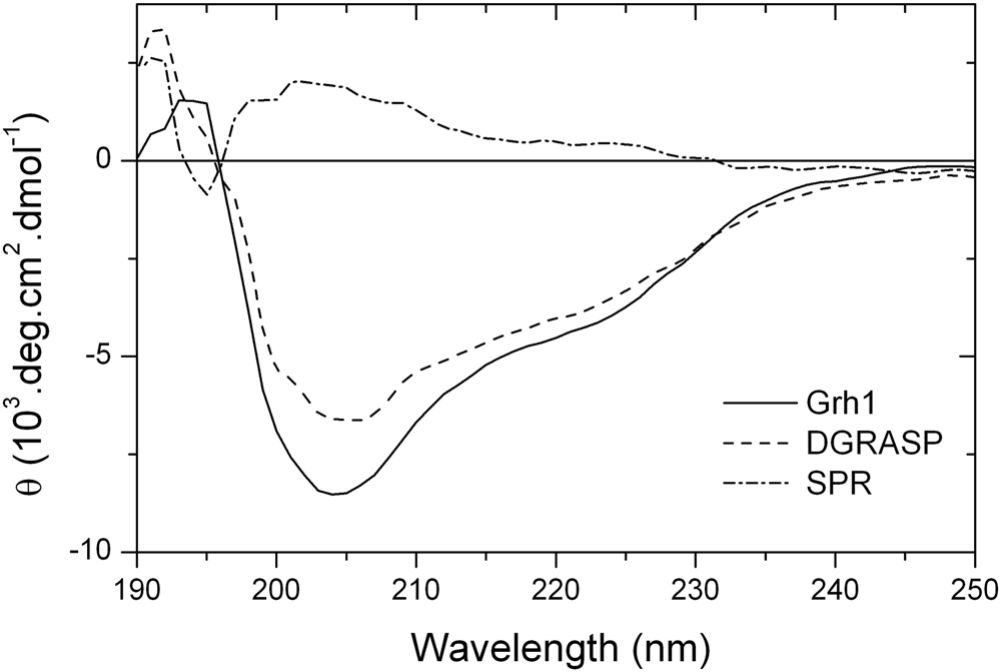
Far UV CD spectra of Grh1 (solid line), DGRASP (dotted line) and the SPR domain (dash line – Grh1 subtracted of DGRASP).

The CD spectrum of Grh1 in aqueous solution (Fig. 3) has a minimum around 204 nm and a poorly resolved and lower intensity peak at 222 nm, which are features typical of CD spectra of proteins with a high content of unordered structures^19^. However, the negative peak at 222 nm is an indication of some ordered elements. Although the intensity ratio of the peaks at 222 nm ([−4,264 deg.cm^2^.dmol^−1^) and 200 nm (−6,904 deg.cm^2^.dmol^−1^) in the CD spectrum of Grh1 is similar to values observed for other proteins in the pre-molten-globule-like state, according to the “double wavelength” plot, [θ]_222_ vs. [θ]_200_^19^, Grh1 does not fit perfectly as a natively unfolded protein based on the estimation of its secondary structure content (11.5% α-helix, 22.1% β-sheet, 17.4% turns, and 49.8% random coil). Comparing these results with those from the GRASP domain only, we observe that here the spectrum also presents a minimum around 200 nm and a low (even lower than for the whole protein) intensity peak at 222 nm, which suggests decreased ordering of the protein structure (Fig. 3). In fact, when we subtract the DGRASP spectrum from that of Grh1, we have a spectrum that resembles that of a Poly(Pro)II conformation^34^, which is expected based on the high content of prolines in the SPR domain.

### Effects of Strong Denaturants

**Figure 4:**
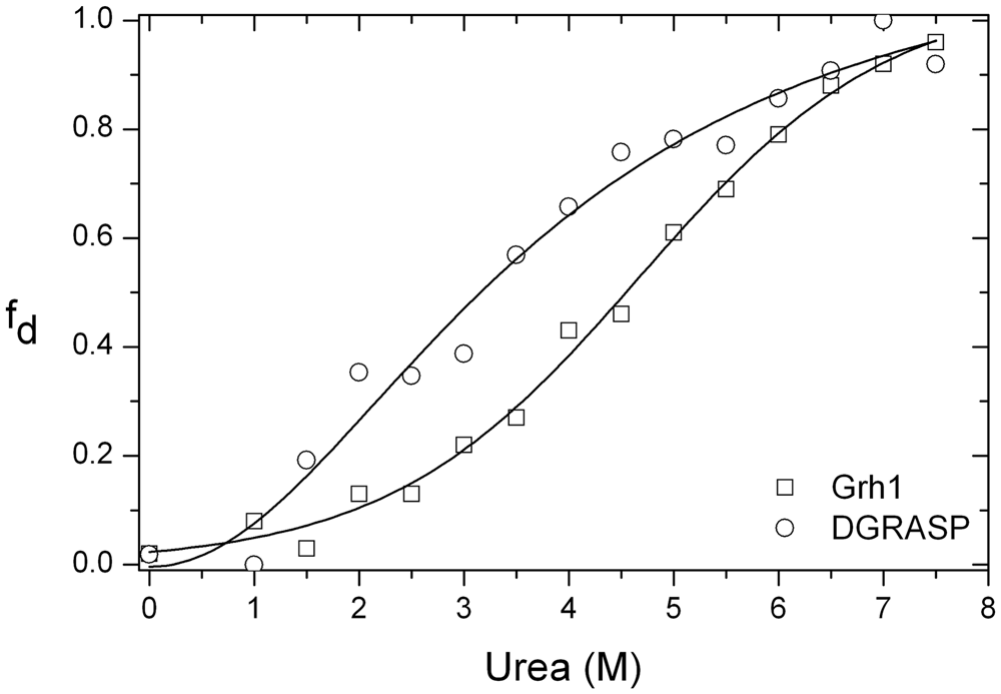
The unfolding fraction (f_d_) of Grh1 and DGRASP obtained from the CD intensity at 222 nm upon increasing concentrations of the denaturant. The solid lines are fits of a Boltzmann model to the experimental data.

The urea-induced unfolding of Grh1 and DGRASP were analyzed by CD and fluorescence spectroscopies. The unfolding monitored by CD spectroscopy (Fig. 4) is a low cooperative transition as seen in the gradual change of the denatured fraction of the protein (f_d_) calculated from the molar ellipticity at 222 nm. The sigmoid-like transition is not as abrupt as expected for well-structured proteins of similar size^19^. The low steepness of the transition curve is typical of native molten globules or native coiled proteins and is due to the low percentage of secondary structure^19,31^. We also obtain a low cooperative unfolding pattern for DGRASP, suggesting that the pattern observed for Grh1 does not come only from contributions of the SPR domain, but also from the GRASP domain (see below).

**Figure 5:**
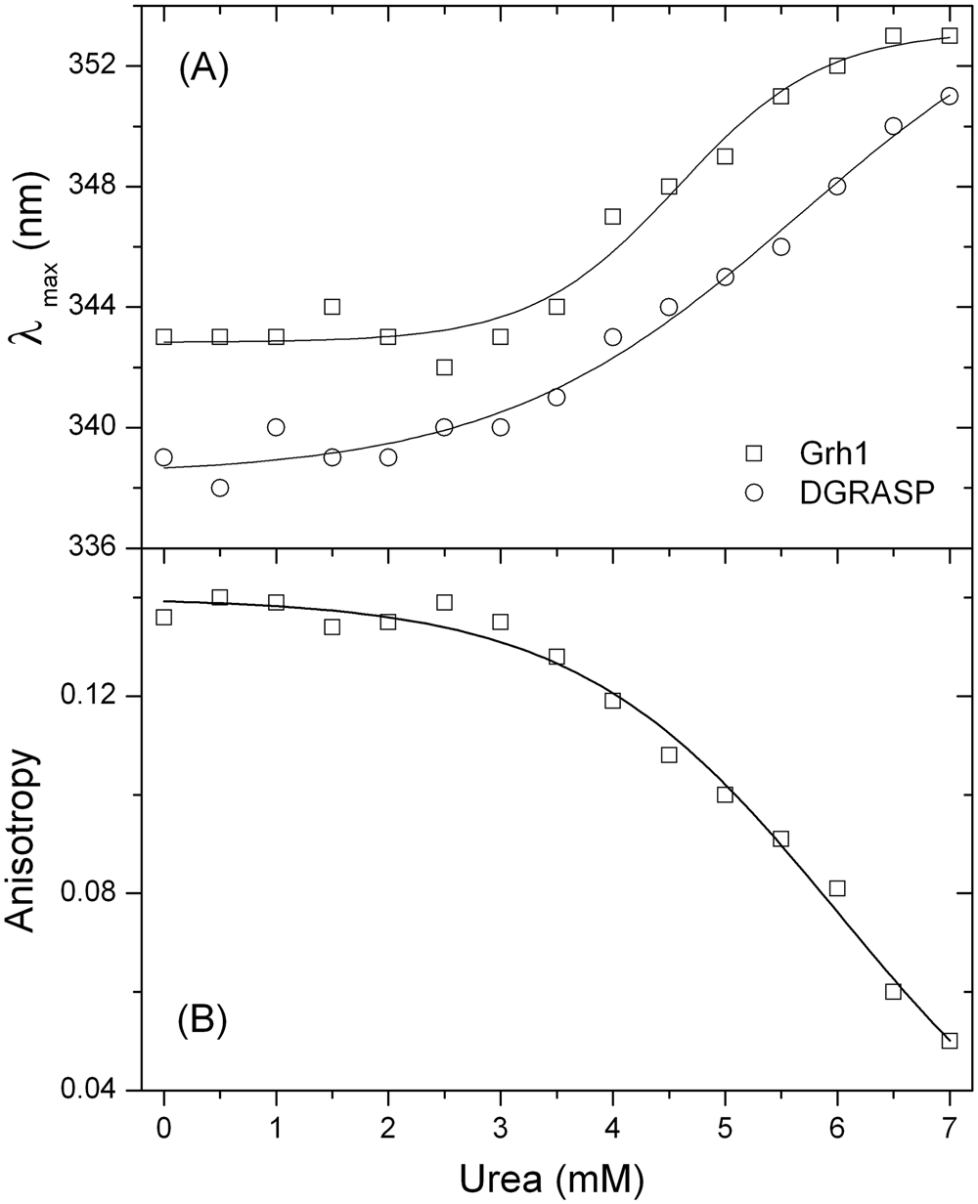
(**A**) Changes in the emission maximum (λ_max_) and (**B**) in the steady state anisotropy of Trp fluorescence as a function of the denaturant concentrations. The solid lines are fits using a sigmoidal Boltzmann function.

The urea-induced unfolding was also monitored by using the wavelength of maximum fluorescence emission (λ_max_) of the tryptophan residues. Tryptophan in the aqueous environment has its maximum fluorescence emission around 350 nm, which is shifted to 320 nm when the aminoacid is placed in the hydrophobic core of proteins^35^. For Grh1 in solution, the λ_max_ is centered at 344 nm indicating the tryptophan residues are exposed to the solvent. The fluorescence signal shows a red shift, in a cooperative transition, from 344 to 352 nm upon increasing urea concentrations (Fig. 5A), indicating further exposure of the tryptophan residues and complete loss of the protein structure. Furthermore, at low concentrations of urea, the fluorescence anisotropy values remain unchanged up to a concentration of 2.5 M, dropping then significantly from 0.14 to 0.05 when urea concentration increases to 7 M, thus suggesting a relevant decrease of the structural ordering around the tryptophan residues during urea denaturation (Fig. 5B).

Since the three tryptophans present in Grh1 are found in the PDZ2, the same fluorescence experiments performed with DGRASP give similar results. However, in this case, the λ_max_ is at 339 nm, a slightly lower value than for Grh1, indicating that the tryptophan residues are less exposed to the solvent as compared to the whole protein. The presence of the SPR domain in the full-length protein seems to induce higher exposure of the inner regions of the PDZ2 domain, suggesting that they do not form two completely separated unities and they may, somehow, interact with each other.

Our observations of the urea-induced unfolding of Grh1 and DGRASP show weak cooperative transitions monitored by CD and somewhat more cooperative unfolding when looking at Trp fluorescence. This apparently disagreement can be explained by the origin of the chromophore under investigation in each method. Far-UV CD measures the optical activity originated from the peptide bonds, whereas fluorescence detects the emission of light generated by specific residues in the protein structure (in our case, Trp residues). We can thus see that CD is reporting unfolding of the overall protein structure, while Trp fluorescence is telling the same story from a more localized point of view. The differences in cooperativity seen from those methods indicate the coexistence of disordered and ordered regions both in Grh1 and in DGRASP, which is in agreement with our CD deconvolution and disorder prediction results (see above). The features observed so far, including low protein compaction but still significant amount of ordered secondary structure and low cooperativity during the unfolding transition, are characteristic of molten globule structures, a behavior already observed for a Grh1 homologue^31^. Interestingly, the SPR domain does not seem to be determinant for this, which is an issue still to be addressed in further details. Because the GRASP domain is the most conserved region within the GRASP family^6^, we can strongly suggest that members of this family might all be molten globule-like proteins.

### Effects of organic solvents

Based on the results shown in the previous sections, we conclude that Grh1 behaves as a molten globule like protein in solution and presents features attributable to proteins containing multiple intrinsically disordered regions. It has been shown that GRASP from *C. neoformans* (CnGRASP) experiences multiple disorder-to-order transitions upon changes in the dielectric constant of the medium or dehydration^36^. GRASPs are peripherally associated to membranes, so it is expected that disturbances in the physicochemical parameters induced by biological membranes may have some influence on their structure. A unique disturb induced by the biological membrane is the change in the dielectric constant (ε) nearby its surface^37,38^. Typically, a dielectric gradient is observed at the membrane/water interface, which can be modeled by an exponentially increasing function from ε = 2–4 at the first water layer up to 78 at approximately 5–6 nm from the interface^38^. In order to check whether Grh1 is also affected by those alterations in the medium, we performed CD experiments in the presence of organic solvents as mimetic models for the ε variation.

**Figure 6:**
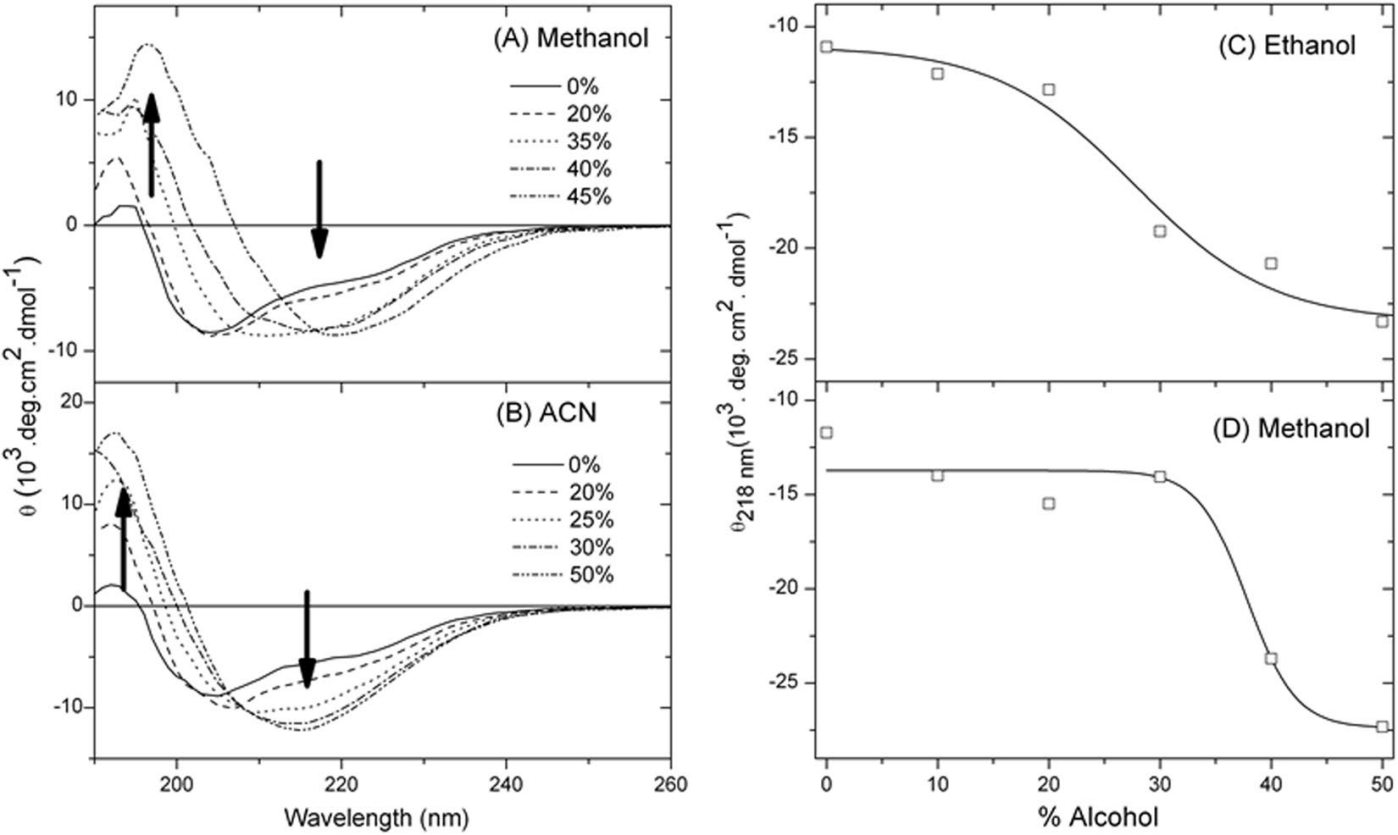
Far-UV CD spectra monitoring the effects of (**A**) methanol, and (**B**) ACN on Grh1 structure. Molar ellipticities at 218 nm upon increasing concentrations of (**C**) ethanol and (**D**) methanol.

Figure 6 shows that the shape and intensity of the CD spectrum of Grh1 considerably change in the presence of non-aqueous solvents manifested by the increase in the negative ellipticity around 222 nm. As observed in Table 1, the content of helical structure increases 43% and reaches a maximum in 35% methanol solution. Grh1 behaves similarly to CnGRASP up to this methanol concentration^36^. For further increases in methanol, a distinct pattern is observed: Grh1 gains β–sheet secondary structure and loses disordered regions as methanol increases (Fig. 6A). The disordered regions decreased 41% in 45% methanol solution. A similar behavior is observed with high concentrations of ACN that induces β-sheet (23%) and helical (51%) conformations and reduces in 50% the disordered regions (Table 1 and Fig. 6B). Hence, the decrease in ε induces the collapsed intrinsically disordered Grh1 to fold in a multiphasic manner, just as described by Uversky^19^ for α-synuclein.

**Table 1:** Secondary Structure Content of Grh1 as obtained from deconvolution of the respective CD spectra.

(A) MeOH	α-helix	β-sheet	Turn	Disordered	NRMSD	(B) ACN	α-helix	β-sheet	Turn	Disordered	NRMSD
0%	0.12	0.22	0.17	0.49	0.067	0%	0.12	0.22	0.17	0.49	0.067
10%	0.14	0.22	0.16	0.47	0.099	10%	0.16	0.26	0.18	0.40	0.134
20%	0.17	0.25	0.18	0.41	0.134	20%	0.20	0.24	0.20	0.37	0.073
30%	0.17	0.25	0.19	0.39	0.100	25%	0.22	0.26	0.23	0.30	0.086
35%	0.20	0.26	0.21	0.33	0.093	30%	0.24	0.26	0.23	0.27	0.077
40%	0.17	0.32	0.21	0.30	0.072	40%	0.23	0.28	0.24	0.25	0.074
45%	0.15	0.33	0.23	0.29	0.093	50%	0.23	0.29	0.24	0.24	0.077

The deconvolutions were performed using the Dichroweb software, with the k2d algorithm^58^. The data refers to CD spectra measured in increasing concentrations of (A) MeOH and (B) ACN.

In all cases, the CD spectra at the end of the organic solvent variation show a pronounced minimum in the vicinity of 218 nm, typical of folded proteins with β-enriched structures. One can see the transition from α-helical (0% alcohol) to β-rich structures (50% alcohol) in ethanol and methanol (Fig. 6C,D) as monitored by changes in the ellipticity at 218 nm. Uversky^19^ described a similar observation when investigating the formation of oligomers of α-synuclein. Considering the fibrillar behavior of α-synuclein depending on the environment^19^ and, being the formation of the β-rich Grh1 irreversible, we hypothesized that the gaining of β-sheet structure could, in fact, be also associated with the formation of fibrils.

### Effects of Temperature

**Figure 7:**
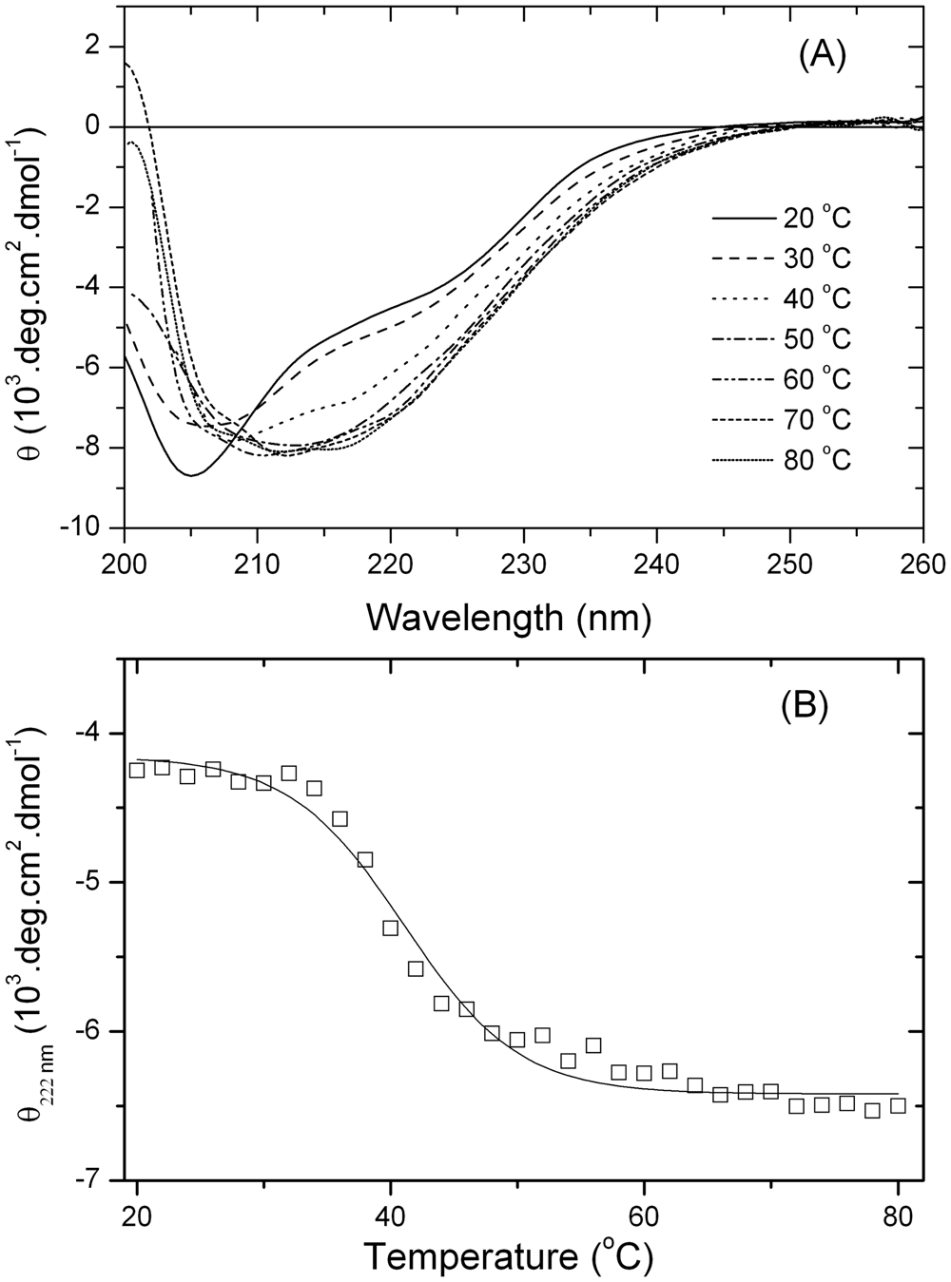
(**A**) Far-UV CD spectra of Grh1 upon heat-induced unfolding in aqueous solution from 20 to 80 °C. (**B**) Thermal unfolding monitored by the molar ellipticity values at 222 nm.

Far-UV CD was also used to analyze the thermally induced unfolding of Grh1. Figure 7 represents the far-UV CD spectra of Grh1 measured at different temperatures and shows that the Grh1 spectrum has its shape significantly changed as a function of the temperature. However, the spectra at higher temperatures are not typical of unfolded structures as observed in the thermal unfolding of globular proteins^39^. Instead of reaching a completely unfolded state, Grh1 irreversibly transitioned to a conformation still showing high contents of secondary structure. As the temperature is increased, the minima at 222 nm and at 205 nm become more and less intense, respectively, yielding a mid-point melting temperature (Tm) of 39.1 °C (Fig. 7B). Interestingly, it has been previously observed a quite similar result for extended IDPs^19,40^, where it has been proposed that the hydrophobic interactions at higher temperatures are the driving forces for the folding of the polypeptide chain. However, in the previous cases there is a transition from a fully unfolded state to a still unfolded one but with a small increase in helical content, whereas for Grh1 there is a “shape shift” from a folded conformation to a final unknown conformation, which is still rich in ordered secondary structure. Interestingly, the far UV CD spectra progressively undergo a shift to spectra with a minimum at 218 nm, and whose shape and intensity measured at temperatures above 45 °C are close to those recorded in 40% methanol and 50% ACN solutions (Fig. 6A,B), showing a β-sheet enriched conformation. Unlike other IDPs, in which temperature effects are reversible^19^, once Grh1 reaches the β-sheet rich conformation, the structure is no longer changed upon cooling. The results in Fig. 6 and in Fig. 7 suggest that the partial structure disturbances induced by either moderately higher temperature or decrease of ε are sufficient to trigger a transition to an ordered still unknown state of Grh1.

Those observations upon changes in temperature and in the presence of organic solvents indicate that, depending on the environment, Grh1 assumes a transient conformation but above a determined threshold, a β-sheet rich conformation is adopted and changes are no longer observed even at high temperatures (up to 80 °C).

### Aggregation Prediction and Sequence Analysis

**Figure 8:**
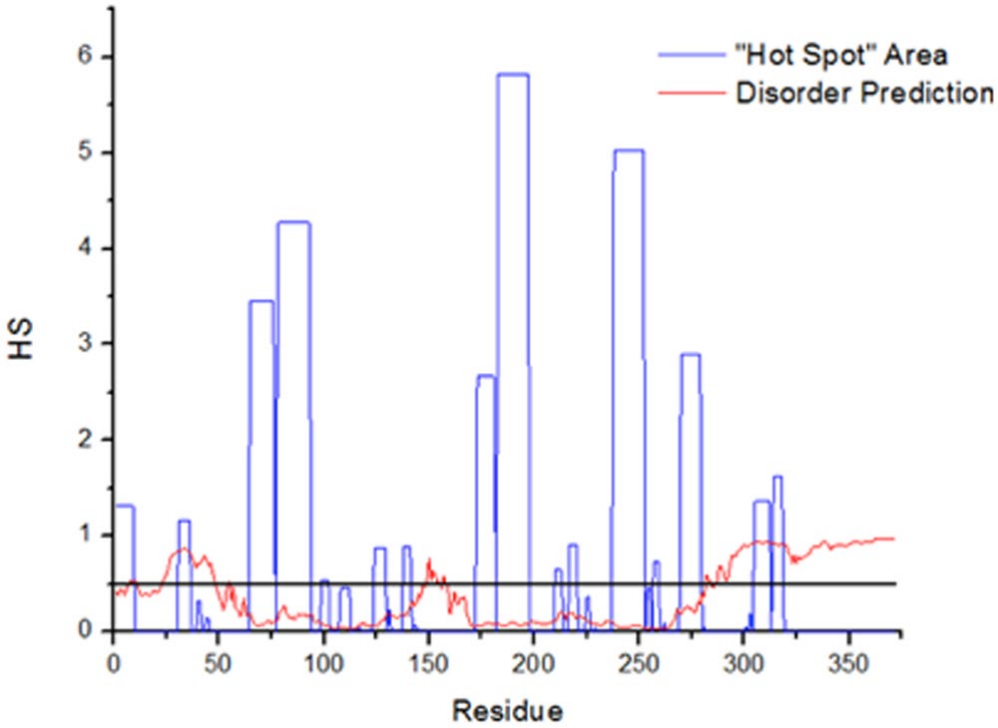
Aggregation prediction for Grh1, done in the AGGRESCAN server, represented by “Hot Spot” areas in blue. The red lines represent one of the disorder predictions showed in Fig. 1

The appearance of considerable β-sheet contributions to the CD spectra of Grh1 either upon heating (Fig. 7A) or in the presence of organic solvents (Fig. 6) prompted us to investigate whether those β-sheet conformations could be related to aggregation. Similar to the prediction of intrinsic disorder, there are now a number of algorithms to predict the protein regions prone to aggregation. One can have information on aggregation propensities by looking at specific residues that are known to be more common in, for example, amyloid fibrils, such as glutamine and asparagine. The server AGGRESCAN^21^ evaluates the protein’s primary sequence, classifying the residues in prone or not prone to aggregation. This classification is based not only on the nature of the residue itself, but also on its surroundings (in our case, we chose a five residue window, which means the residue will be evaluated together with the two previous and the two subsequent residues). With that classification, a Hot Spot (HS), the server creates a region where 5 or more residues are considered to be prone to aggregation. The longest the region and the aggregating nature of the residues, the higher the HS. The aggregation profile of Grh1 is shown in Fig. 8, and we can see a number of short along with three long HS. While the predictor is not exclusive for fibril formation, since other aggregates can exist, it gives a good hint on whether or not a determined region is more likely to form fibrils.

The existence of potential aggregation spots brings the close link between aggregation and intrinsic disorder into play^41^. Hence, to check for correlations between intrinsic disorder and aggregation in the case of Grh1 we also show in Fig. 8 one of the disorder predictions presented in Fig. 1. The black solid line represents the threshold for intrinsic disorder. The flexibility gained with a less compact structure can be used to help overcoming energy barriers needed for the formation of the aggregate. Several structural arrangements of disorder and aggregate-like regions in proteins have been proposed^41^ and in one of them the amyloid core is flanked by intrinsically-disordered regions (IDRs), which could be the geometry adopted by Grh1 as suggested by the intrinsically disorder and aggregation propensities shown in Fig. 8.

As for the final residues in the sequence, those in the SPR domain, it is reasonable not to observe aggregation since prolines are considered to be chain breakers, thus leading the score of a determined window in AGGRESCAN to 0. That means a domain such as the SPR would not aggregate. We can also think of that in terms of the structure of the fibril: to accommodate a proline into a β-sheet is very costly in terms of energy^42^.

### Ghr1 forms β-sheet rich amyloid fibers

#### 8-anilino-1-naphthalenesulfonic acid (ANS) assay

Our bioinformatics analysis strongly suggested that Grh1 contains regions that are prone to aggregation, which, in conjunction with our results on the intrinsically disordered nature of part of Grh1 structure, indicate that Grh1 would be able to form β-sheet rich amyloid fibers. The formation of fibrils is a process that includes the formation of small oligomers that associate due to a destabilization of the native structure, leading to the formation of a number of partially folded intermediates, which possess increased aggregation propensity. This process is often called “monomer activation”^43^. In their review on the modeling of amyloid fibril formation, Gillam and MacPhee^44^ cover the first moments of amyloid formation, called the lag phase, the mechanisms underlying the growth phase, where the formation of the proto-fibrils happens, until their assembly in amyloid fibrils, on the plateau phase. If we look at the whole process during time, we will have a sigmoid-like behavior much like the one we see in our CD experiments (Fig. 6C,D).

**Figure 9:**
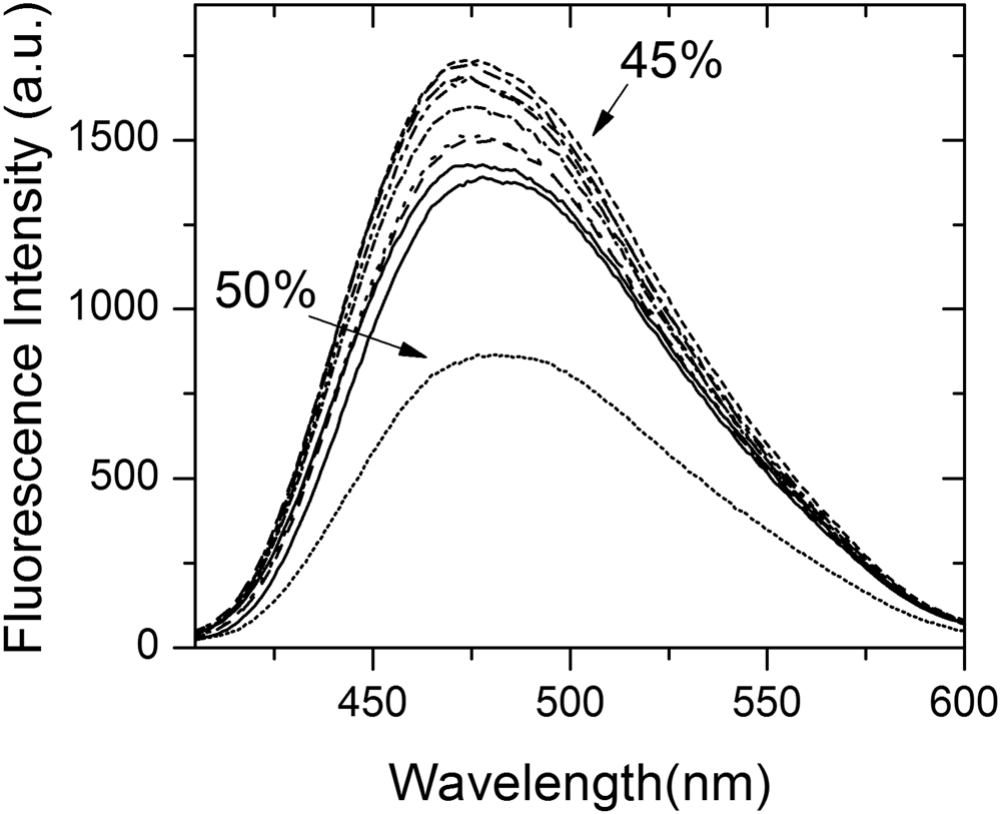
Fluorescence spectra of ANS bound to Grh1 with increasing concentrations of ethanol. The arrows point to the spectra in 45% and in 50% ethanol, emphasizing the reduction in intensity above 45% ethanol.

To further investigate if Grh1 is really forming fibers depending on the environment conditions, we followed the well-established protocols based on the use of the fluorescence of extrinsic dyes^42^. ANS is a fluorescent dye commonly used in protein folding studies^45^. Although it is not specific for amyloid fibrils, the experiment we conducted followed previous studies related to fibril formation. Bolognesi *et al*.^42^ were able to trace all the phases of fibril formation as a function of increasing concentrations of a fibril trigger. Even more interesting in that report, the authors were able to establish a good relation between ANS fluorescence and the presence of proto-fibrils. Since ANS will bind to accessible hydrophobic cores in the protein, when the monomers assemble into proto-fibrils, there will be new hydrophobic sites created, thus increasing ANS fluorescence intensity. Keeping the stimulus by increasing the trigger concentration, the system is forced into the plateau phase, where the proto-fibrils assemble to form the proper amyloid fibrils. By doing so, the fibrils lose hydrophobic sites previously present, and then the ANS fluorescence decay^42^. Figure 9 shows how the ANS fluorescence will increase with increasing concentrations of ethanol (that we had seen on the CD experiments to lead to aggregation), until it reaches a maximum in 45% ethanol, and then decreases in 50% ethanol, which is in agreement with our CD data (Fig. 6C). Although we cannot see the sigmoid-like time-course formation of the fibrils, we have data that is consistent with previous findings regarding ANS binding to proto-fibrils (inset in Fig. 9).

### Thioflavine T (ThT) assay

**Figure 10:**
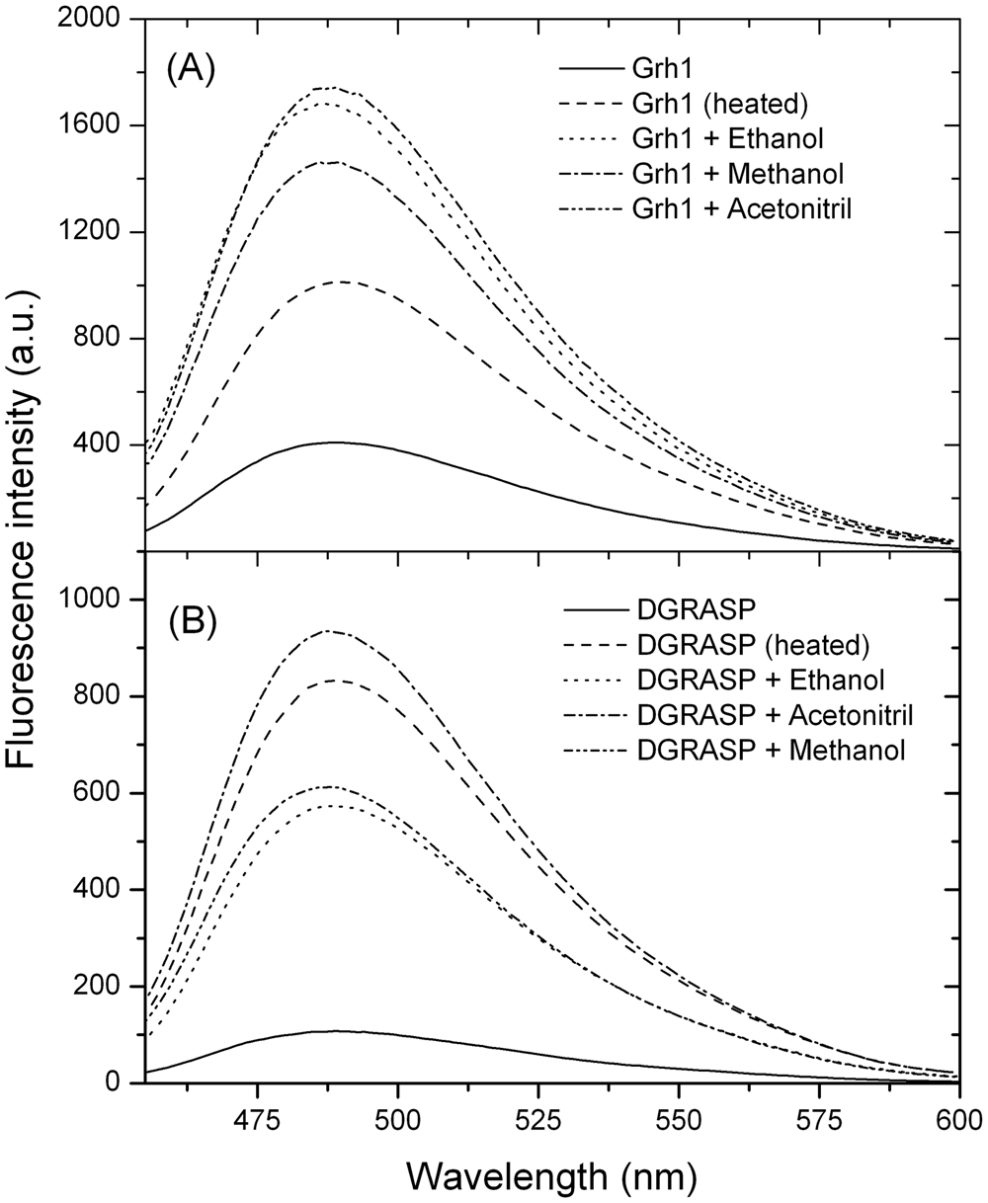
Fluorescence spectra for ThT bound to (**A**) Grh1 and (**B**) GRASP domain only, in different conditions.

The ANS assay described in the previous section indicates that Grh1 undergoes a structural transition from monomers to fibrils upon increasing ethanol concentration. To check whether those fibrils present amyloid features, we performed an assay based on the use of the fluorescence of Thioflavine T (ThT) in the presence of Grh1. ThT is a fluorescent dye used in the detection and characterization of amyloid fibrils *in situ*^29^. It works as a fluorescent rotor that binds into β-sheet cavities^46^. When in solution the fluorescence is weak due to ThT freedom of rotation. When bound to fibrils, there is less torsional relaxation, leading to an expressive increase in fluorescence^29^. Although ThT can bind to amorphous aggregates and other structures with minor affinity, it is considered specific for amyloid fibrils^29^. In Fig. 10A, we see weak fluorescence when ThT is in solution with Grh1 in its native form. However, when ThT is in solution with Grh1 previously submitted to the conditions we have seen to induce aggregation (we tested for temperature, methanol, ethanol and acetonitrile) there is at least a 10-fold increase in the fluorescence intensity. We tested our construction without the SPR domain (Fig. 10B) in the same conditions and we observe the same increase in ThT fluorescence. Although the comparison between the ThT data for Grh1 and the GRASP domain cannot provide any insight into the route of fibril formation, it is nevertheless another proof that the SPR domain is not needed for the fibrillation to occur. Furthermore, we can see that different conditions led to different intensities in ThT fluorescence. For both Grh1 and DGRASP, ACN showed to induce the largest change, while heating led to a large change in DGRASP, but a not so pronounced one for Grh1, in which the effects of ethanol and methanol were markedly more pronounced. That could be the result of either the preparation of the samples not being exactly equal, or it could be related to the pathways and the configuration that each condition induced.

### Congo Red (CR) assay

**Figure 11:**
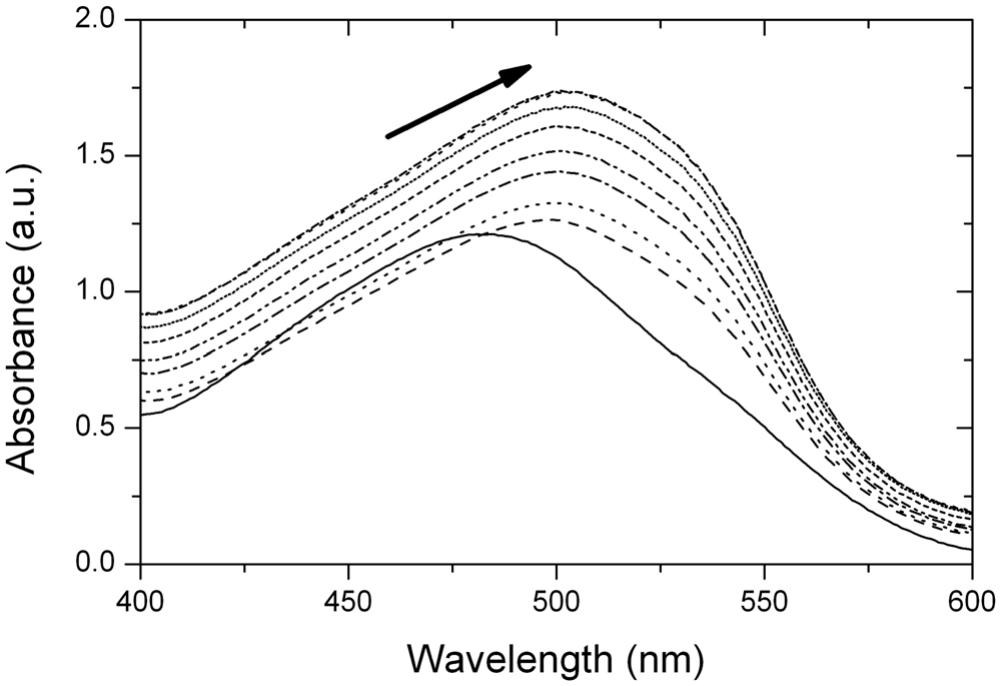
Absorbance spectrum for Congo Red free and bound to pre-heated Grh1.

CR is another widely used dye to probe amyloid structures^29^. The exact mechanism of binding is still unknown, but there are some models for it, such as ionic interactions between the sulfonate group of CR and basic residues in the aggregate^47^. It is possible to use the birefringence of the amyloid fibrils with CR to prove their existence but, since several participants are inherently birefringent (such as buffer salts), the technique is quite subjective and requires a known amyloid structure as control^29^. For that reason, we chose another approach based on a spectrophotometric assay. For this experiment we used pre-heated Grh1 to 50 °C to assure fibrillation. In Fig. 11 we can see that the absorbance of CR between 400 and 700 nm increases linearly with the increase in pre-heated Grh1. We tested for the native form of Grh1, but there is no change in CR absorbance, showing that the binding only takes place with the protein in its fibrillar (pre-heated) form. Despite the fact that CR binding is one of the most accepted evidences of amyloid formation, it is now known that amyloid fibrils of different compositions may bind to CR through different mechanisms, which can change CR response^29^. The most pronounced change in absorbance intensity around 540 nm (in this experiment, the largest difference was in 533 nm) is believed to be characteristic of amyloid fibrils.

### Kinetics of fibril formation

To check fibril formation as a function of time, we used the strategy presented by Chan *et al*.^48^, in which the authors use the so-called fibril intrinsic fluorescence in the visible range. Chan *et al*.^48^ discuss the properties that allow a protein in its fibrillar state to fluoresce, while this is not seen with the protein in its native form. The authors suggest that it is the delocalization of electrons via multiple bond conjugation, present in β-sheet rich structures, that gives rise to the fluorescence emission in the visible range. This intrinsic fluorescence in the visible range has been used to give insights on fibrillation modes^48,49^.

**Figure 12:**
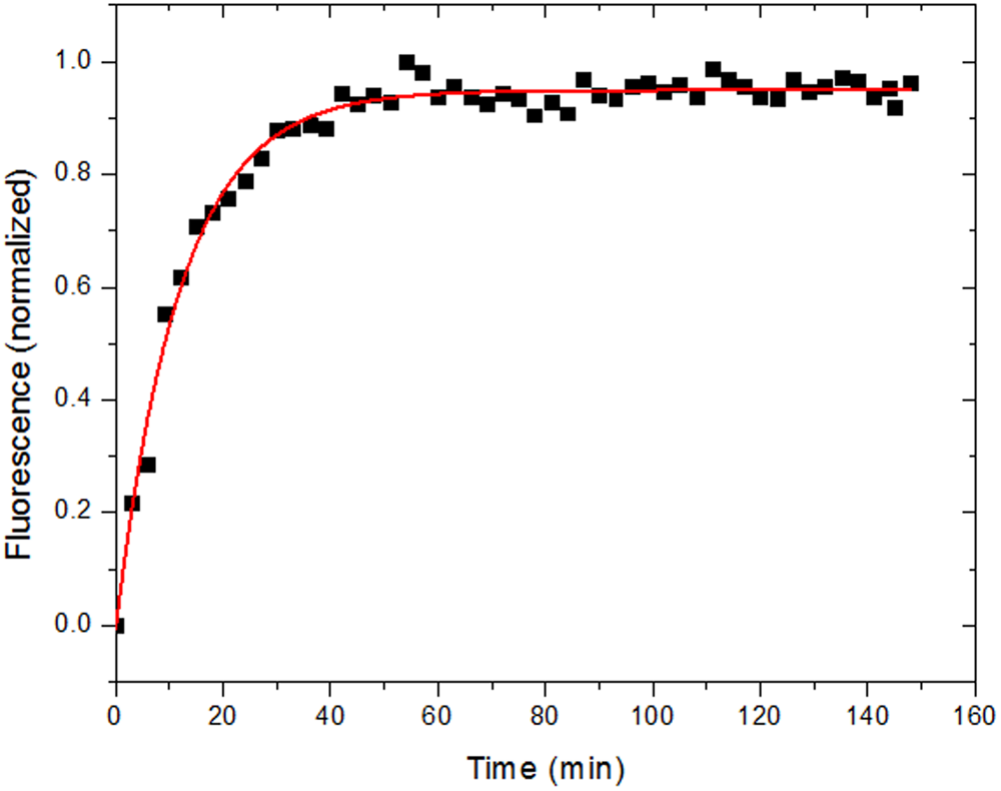
Normalized intrinsic fluorescence of Grh1 heated to 50 °C, as a function of time. Excitation wavelength: 357 nm Emission wavelength: 470 nm.

We then followed that strategy by monitoring the fluorescence intensity of Grh1, when submitted to high temperature (50 °C), as a function of time and the result can be seen in Fig. 12. Although a lag phase does exist, it is probably too fast to be detected in this manner. This suggests the most suitable fibrillation model in our case is the one described by Kumar *et al*.^50^, with the best fitting mode being asymptotic, which accounts for a nucleation independent pathway, rather than sigmoidal.

**Figure 13:**
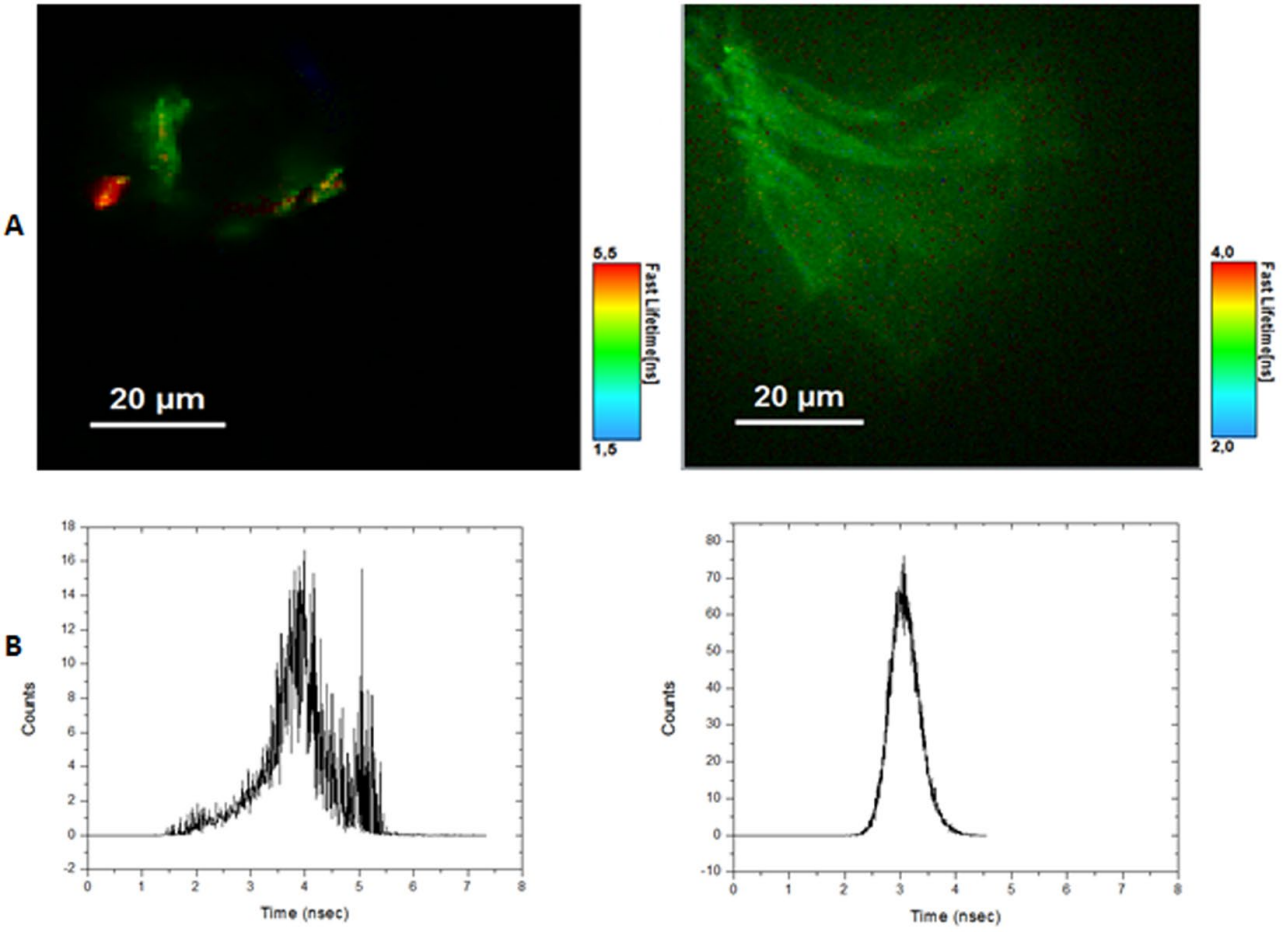
Results of the FLIM experiments. (**A**) Lifetime Microscopy Images of 15 μM samples and (**B**) Fluorescence Lifetime Histograms for samples heated for 30 minutes, on the left, and 90 minutes, on the right.

Based on the kinetics shown in Fig. 12, we can see that the fibrillation process takes ca. 25 minutes to reach the plateau phase. To actually see the fibril formation and respecting such constraint, we decided then to use the Fluorescence Lifetime Imaging Microscopy (FLIM), relying once more on the intrinsic fluorescent properties of the fibrils. While this technique does not tell us anything about the size of fibrils, the lifetime measurements can be of help in the matter of deciding whether we have protofibrils or grown fibrils. We ran controls with Grh1 in its native state, and no fluorescence (as expected) was detected (data not shown). On the other hand, upon sample heating for 30 and 90 minutes, we could clearly detect the intrinsic fluorescence of Grh1 (Fig. 13).

In Fig. 13, we also present the histograms corresponding to the fluorescence lifetimes of the populations giving rise to the images in the upper panels of the figure. The images in Fig. 13A as well as the respective histograms in Fig. 13B show that the heating of the sample for 30 minutes produced a more heterogeneous distribution of fluorescence lifetimes than for the 90 minutes of heating. The presence of different colors in the left panel of Fig. 13A clearly indicates the presence of a broader distribution of lifetimes, which is corroborated by the corresponding histogram. After 90 minutes, the particles gave rise only to green spots in the image. More quantitative information can be observed in Fig. 13B: the lifetimes are longer and broader for samples heated up to 30 minutes, while within 90 minutes there is a more homogeneous distribution, and on average a shorter lifetime. These data are in agreement with what is described by Chan *et al*.^48^, whose work showed a lifetime of fluorescence of 2 to 4 nanoseconds for amyloid fibrils, which is the value found in our experiments.

## Conclusions

In this manuscript, we have described a biophysical characterization of Grh1 and its GRASP domain that revealed two significant aspects about Grh1, which are most likely linked: the presence of multiple intrinsically disordered regions that confers to Grh1 a molten globule-like feature and the capability of forming amyloid fibrils upon mild denaturing conditions, in an SPR-independent fashion. Grh1 structural dynamics in solution seems to be high but still showing a minimum stable tertiary structure. However, when a destabilizer condition, such as high temperature and/or the membrane surface, is introduced and the structure is slightly disturbed, an irreversible transition associated with amyloid fibril formation is induced. Interestingly, amyloid formation of b2m, a dialysis-related amyloidosis disease resulting from deposition of amyloid aggregates in skeletal tissue, is strongly enhanced in conditions that destabilize its globular structure^51^. Besides, the interaction between α-synuclein and lipids has been also shown to modulate amyloid fibril formation, depending on the relative proportion of the two species^52^, suggesting that the membrane surface is capable of triggering fibrillation. It has been suggested that partially folded α-synuclein structures induced by increasing temperature is stabilized by self-assembly and that these oligomers may evolve into the fibril nucleus, besides having all the properties expected for a molten globules^5^. In general, misfolding intermediates play a key role in defining aberrant protein aggregation and amyloid formation in several different human diseases^5^. We observed that the GRASP domain is capable of forming fibbers in a SPR independent way, and since this is the most well conserved region along GRASP family, it is reasonable to expect the same amyloidogenic pattern for all members.

Amyloid fibrils are also found in a diversity of organisms, such as plants and bacteria^41^, and *Saccharomyces cerevisiae* is not an exception. There are reports of amyloid proteins in yeast, such as the termination factor Nab3, that together with other two proteins forms a complex that is the major termination tool for short, non-coding RNAs^53^.

Once thought to be disease-related only, today the idea of functional amyloids is widely accepted. Bacteria and even humans can use the properties of some fibrils to perform functions in the organism^54^. Such is the case of Sup35p: yeasts carrying the aggregated form of the protein have selective growth advantage^55^. In the case of Grh1, interestingly, only under growth it localizes to ER exit sites and early Golgi membranes, and the yeast stops growing above 37 °C^55^, around the same temperature we determined that Grh1 forms fibrils. Upon stress conditions, like starvation and incubation at the non-permissive temperature of 37 °C, Grh1 redistributes normally to a large compartment called compartment for unconventional protein secretion^56,57^. Thus, a hypothesis for further investigation is whether the formation of fibrils by Grh1 takes part in membrane fusion events to help generating compartments involved in unconventional secretion^56^. Because we observed that both temperature and the membrane surface could affect the fibril formation, it is interesting to explore deeply the phenomenon when both perturbations are present together. Experiments to address this are currently being performed.

Understanding the relationships between protein structure and function is one of the fundamental questions in molecular biophysics. We proved that Grh1 is a marginally stable protein and undergoes folding reactions that involve different kinds of ordered forms depending on the environment. The functional diversity reported for Grh1 can then greatly benefit from the possibility of becoming more ordered or folded into stable secondary or tertiary structures and increase the specificity of binding. Furthermore, the irreversible quaternary structure it adopts (the amyloid fibrils) in some conditions might be a strategy of evolution to help survivability in undesired conditions.

## Electronic supplementary material

Supplementary figure

## Data Availability

All data generated or analyzed during this study are included in this published article and in its Supplementary Information.
