# Supplementary material for: The yeast GRASP Grh1 displays a high polypeptide backbone mobility along with an amyloidogenic behavior
Source: Sci Rep. 2018 Oct 24;8:15690. doi: 10.1038/s41598-018-33955-1 (PMC6200761; doi:10.1038/s41598-018-33955-1)
Supplement: Supplementary file 1 — Supplementary figure [file 41598_2018_33955_MOESM1_ESM.pdf]

The yeast GRASP Grh1 displays a high polypeptide backbone mobility along with an amyloidogenic behavior

Fontana, N. A., Fonseca-Maldonado, R., Mendes, L.F.S., Meleiro, L. P. , Costa-Filho, A. J.

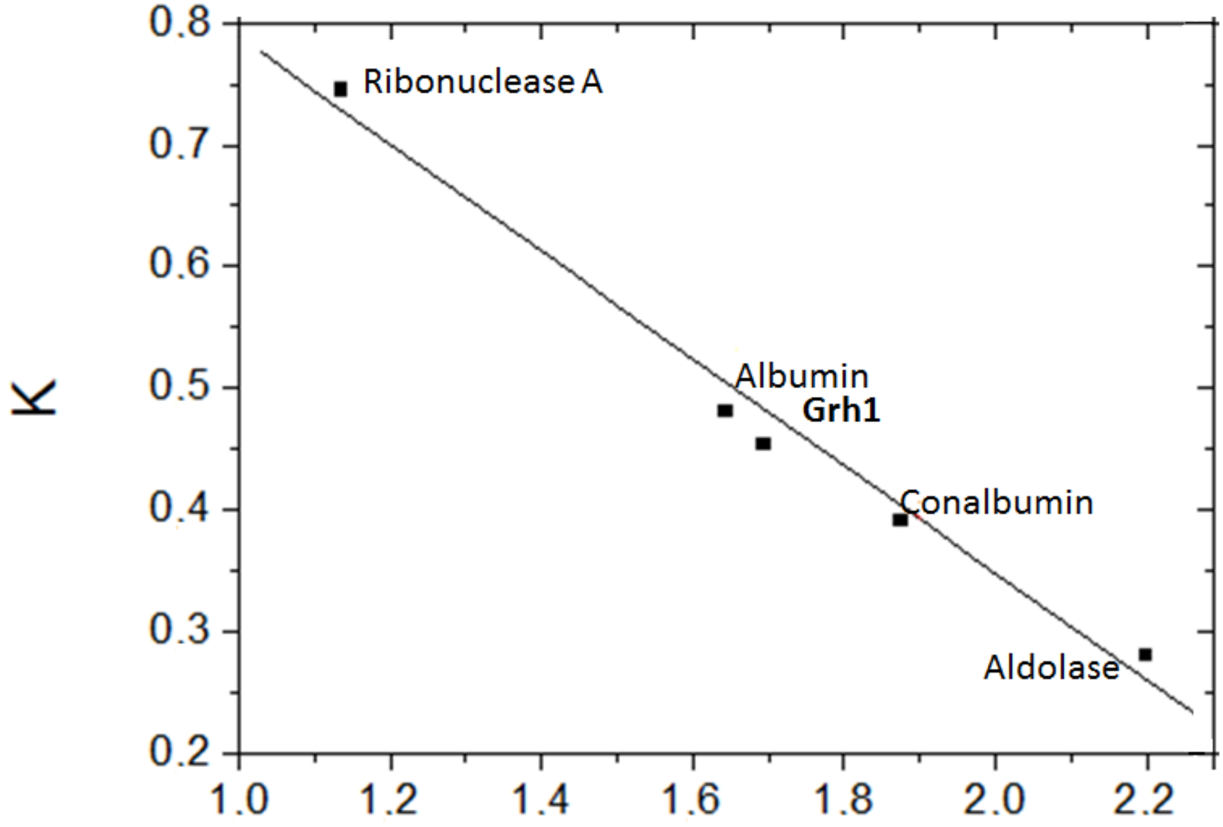

Suppl. Material _Figure 1: Elution curve. Relationship between the partition coefficient (K) and the logarithm of Molecular Mass
